# Synthetic *Brassica napus* L.: Development and Studies on Morphological Characters, Yield Attributes, and Yield

**DOI:** 10.1100/2012/416901

**Published:** 2012-06-04

**Authors:** M. A. Malek, M. R. Ismail, M. Y. Rafii, M. Rahman

**Affiliations:** ^1^Plant Breeding Division, Bangladesh Institute of Nuclear Agriculture, Mymensingh 2202, Bangladesh; ^2^Institute of Tropical Agriculture, Universiti Putra Malaysia, 43400 Serdang, Malaysia; ^3^Department of Plant Science, North Dakota State University, Fargo, ND 58108-6050, USA

## Abstract

*Brassica napus* was synthesized by hybridization between its diploid progenitor species *B. rapa* and *B. oleracea* followed by chromosome doubling. Cross with *B. rapa* as a female parent was only successful. Among three colchicine treatments (0.10, 0.15, and 0.20%), 0.15% gave the highest success (86%) of chromosome doubling in the hybrids (AC; 2*n* = 19). Synthetic *B. napus* (AACC, 2*n* = 38) was identified with bigger petals, fertile pollens and seed setting. Synthetic *B. napus* had increased growth over parents and exhibited wider ranges with higher coefficients of variations than parents for morphological and yield contributing characters, and yield per plant. Siliqua length as well as beak length in synthetic *B. napus* was longer than those of the parents. Number of seeds per siliqua, 1000-seed weight and seed yield per plant in synthetic *B. napus* were higher than those of the parents. Although flowering time in synthetic *B. napus* was earlier than both parents, however the days to maturity was little higher over early maturing *B. rapa* parent. The synthesized *B. napus* has great potential to produce higher seed yield. Further screening and evaluation is needed for selection of desirable genotypes having improved yield contributing characters and higher seed yield.

## 1. Introduction

Allopolyploids are widely spread in the plant kingdom. Their success might be explained by positive interactions between homoeologous genes on their different genomes, similar to the positive interactions between different alleles of one gene causing heterosis in heterozygous diploid genotypes [1]. Amphidiploid species are a form of polyploids that have evolved from interspecific hybridization between two or more diploid species, either through the fusion of unreduced gametes or through interspecific hybridization followed by spontaneous chromosome doubling. Many wild species as well as major field crops like wheat, oat, soybean, cotton, and rapeseed are the result of spontaneous interspecific hybridization, showing the high potential of allopolyploid species.

Allotetraploid *B. napus *(AACC, 2*n* = 38) has evolved from a natural cross between *B. rapa *(AA, 2*n* = 20) and *B. oleracea *(CC, 2*n* = 18) along the Mediterranean coast with uncertain evolutionary origin time approximate ranging from 0.12 to 1.37 million years ago [2, 3]. The short domestication history and traditional breeding schedule of *B. napus* has led to a narrow genetic range in the population. As a whole, although the allopolyploid species has been rapidly and widely cultivated globally as an oilseed due to the advantages of high yield and wide adaptation, rapeseed breeding and heterosis utilization have undergone genetic bottlenecks due to exhaustion of the genetic variation [4, 5]. Artificial synthesis of the naturally occurring amphidiploid *B. napus* by hybridization between its progenitors followed by chromosome doubling provides a means to increase the usable genetic variability [6]. Artificial *B. napus* was also synthesized earlier by Schranz and Osborn [7], Albertin et al. [8], and Gaeta et al. [9]. The present investigation was, therefore, aimed for development of synthetic *B. napus* from the hybrids of its two progenitor species and to study the C_2_ (second colchiploid generation) synthetic *B. napus* compared to its parents regarding some morphological characters, yield attributes, and yield.

## 2. Materials and Methods

The experiments were conducted during November to February each of 2005-2006, 2006-2007, and 2007-2008 at the Bangladesh Institute of Nuclear Agriculture, Mymensingh, Bangladesh.

### 2.1. Plant Materials

Binasarisha-6 of *B. rapa* var. “yellow sarson” and Alboglabra-1 of *B. oleracea* var. “alboglabra” were used as parental genotypes for the development of interspecific hybrids. Interspecific hybrids were induced to double chromosome number for the development of synthetic *B. napus*. Synthetic *B. napus* was compared with parental genotypes.

### 2.2. Crossing and Collection of Hybrid Seeds

Flower buds of each of the female parents, expected to be opened in the next morning, were selected for emasculation. The emasculated buds were immediately pollinated with fresh pollen grains collected from the male parent. Pollinated flowers were covered with thin brown paper bags. The siliqua bearing F_1_ seeds were collected after proper maturation. The hybrid (F_1_) seeds were threshed, dried, and stored for the next season to grow the F_1_ hybrids.

### 2.3. Chromosome Count of F_1_ Hybrid and Pollen Fertility Study

Root tips from the germinating seeds were fixed in acetic alcohol (1 : 3) after pretreatment in saturated aqueous monobromonaphthalene solution for 2.5 hours. The tips were hydrolyzed in 10% HCl for 12 minutes at 60°C and then stained with 1% acetocarmine. Individual chromosome was counted with microscope at 100 times magnification. Acetocarmine (1%) was used for pollen fertility study. Intensely stained and normal shaped pollen grains were scored as fertile while the unstained and collapsed pollen grains were scored as sterile according to Sheidai et al. [10]. The ratio of stained pollen to the total was expressed as percentage of pollen fertility.

### 2.4. Colchicine Application and Development of C_1_ Synthetic *B. napus*

Cotton plug method was followed to double chromosome number in the hybrids. Three concentrations (0.10%, 0.15%, and 0.20%) of colchicine were applied. Colchicine treatments on hybrids were applied at five to six leaves stages. Hybrids grown in pots were placed under shade and the twigs of each hybrid were removed. Two leaf axils of each hybrid plant were selected for treatment. A small cotton wool ball was placed on each of the selected leaf axils. The cotton wool balls were then soaked with colchicine at six hours intervals with 10-microlitre solution following the modified version of Gland [11]. Duration of treatment was maintained for 24 hours. The chromosome-doubled shoots developed from the hybrid plants were named as C_1_ (first colchiploid generation).

### 2.5. Growing of C_2_ Synthetic *B. napus* with Parents and Collection of Data

C_2_ seeds collected from the C_1_ plants having higher percentages of pollen fertility and siliqua setting along with higher number of seeds per siliqua were used for growing C_2_ *B. napus *plants. Parental genotypes were also grown with C_2_ plants in a single replicate in the field. Different cultural practices as well as irrigation and application of pesticides were done as and when necessary for the normal growth and development of the plants. Data were taken with respect to plant height, length and width of petal, number of primary branches per plant, pollen fertility (%), siliqua setting (%), number of siliquae per plant, number of viable and sterile seeds per siliqua, siliqua length, beak length, 1000-seed weight, seed yield per plant, days to flowering, and days to maturity. Data were taken from 40 randomly selected C_2_ plants and 10 randomly selected plants of each parent. Measurements of mean, range, and coefficients of variation (CV%) of each character were calculated following the formula suggested by Burton [12].

## 3. Results and Discussion

### 3.1. Crossing and Study of F_1_ Hybrid

Siliqua and seed setting was fairly good in cross between Binasarisha-6 and Alboglabra-1. Out of 106 crosses of Binasarisha-6 as female parent, 67 siliquae were developed with hybrid seeds and gave 63% success. On the other hand, the reciprocal crosses that is, Alboglabra-1 as female parent, were not successful. These results showed an agreement with Malek et al. [13], Choudhary et al. [14], and Sharma et al. [15] who reported the similar performances between crosses and reciprocals in the interspecific crosses within *Brassica* species. Somatic chromosome number in the hybrids (2*n* = AC⁡) was 19, which showed the equal number of chromosome of amphihaploid between the species, *B. rapa *(*n* = 10, A) and *B. oleracea *(*n* = 9, C). The hybrids exhibited vigorous growth with numerous primary as well as secondary branches. Akbar [16] also observed hybrid vigour in the interspecific hybrids of cross between *B. campestris* and *B. oleracea.* Intermediate morphology of F_1_ in *Brassica* similar to the present study was also reported earlier by Choudhary et al. [17]. Hybrids flowered abundantly having shriveled, pointed tip and pale colour anthers with reduced filaments. Batra et al. [18] also reported similar morphology of anthers in interspecific hybrids within the genus *Brassica*. Hybrids produced 99-100% sterile pollens. Song et al. [19] also observed high pollen sterility in the F_1_ hybrids obtained from all possible combinations of interspecific crosses of the diploid species within the U-triangle. According to Stebbins [20] high pollen sterility as observed in the hybrids of the present study might be due to meiotic irregularities and segregational anomalies as both genomes (A and C) had a single set of chromosome.

### 3.2. Treatment of Dihaploid Hybrids with Colchicine

It was observed that colchicine produced a drastic effect on growing leaf axils. In general, growth and development was strongly inhibited. The treated auxiliary shoots showed very slow growth and development. After three to four weeks of treatment, though growth and development was started, but even then their growth was very slow. The new shoots emerged from the colchicines-treated leaf axils displayed thick and deep green leaves indicating the first symptom of induction of chromosome doubling. The highest chromosome diploidization (84%) was achieved with 0.15% colchicine treatment followed by 0.2% (72%) and 0.1% colchicine gave 60% success. Inhibited growth and development in colchicine treated tissues in *Brassica* hybrids was also reported by Aslam et al. [21]. The results indicated that chromosome diploidization rate differed with the concentration of colchicine, which showed close agreement with the results of Aslam et al. [21]. It has also been reported that success in chromosome doubling differs with method of application, different conditions, duration of treatment, and at different stages of development [21–23]. Chromosome-doubled shoots produced fertile pollens and seeds in the siliquae.

### 3.3. C_2_ *B. napus* and Its Parents

**Figure 1:**
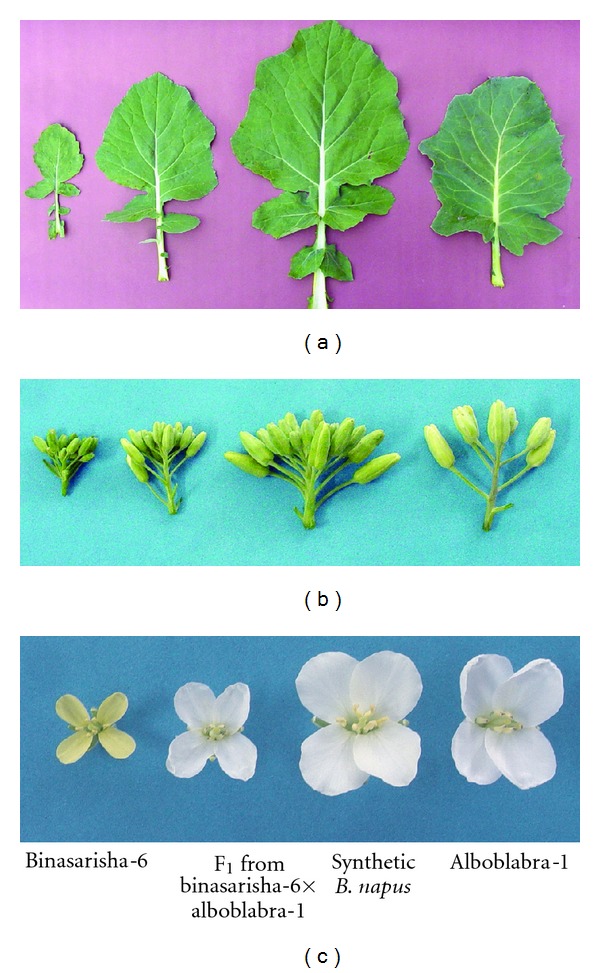
(a) Leaves of Binasarisha-6, F_1_, synthetic *B. napus*, and Alboglabra-1. (b) Racemes of Binasarisha-6, F_1_ hybrid, synthetic* B. napus*, and Alboglabra-1. (c) Flowers of Binasarisha-6, F_1_ hybrid, synthetic* B. napus*, and Alboglabra-1.

**Figure 2:**
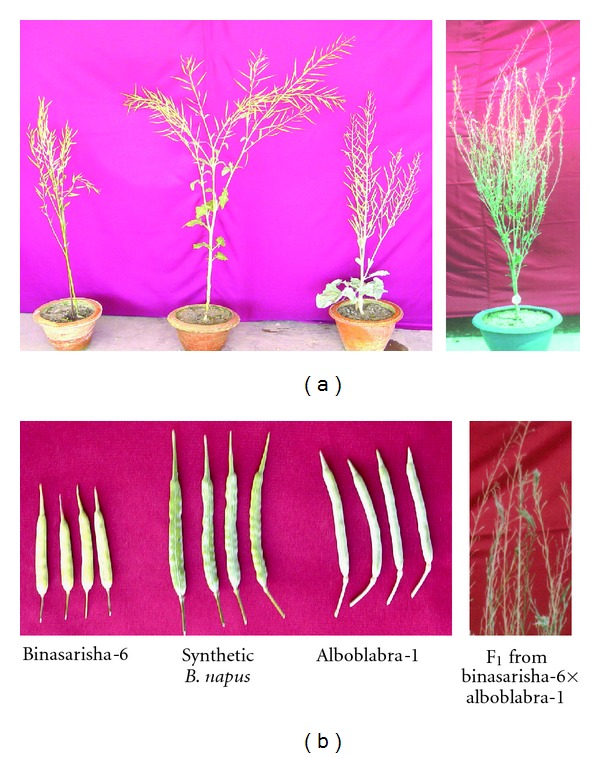
(a) Plants of Binasarisha-6, synthetic* B. napus*, Alboglabra-1, and F_1_, and (b) siliquae of Binasarisha-6, synthetic* B. napus*, and Alboglabra-1, and rachis without siliqua in F_1_.

Colour, shape, and dentition of leaves in C_2_ plants were intermediate between the parents. Leaf size of C_2_  plants was larger than that of both parents and F_1  _(Figure 1(a)). Size of flower buds and flowers of C_2_ plants was also larger than that of both parents and F_1_ (Figures 1(b) and 1(c)). The flowers of hybrids and C_2_ plants had white petals resembling the Alboglabra-1 (Figure 1(c)), which indicates dominance of white petal colour over yellow. Finally, C_2  _plants showed increased vegetative growth over the parents (Figure 2(a)) which agreed to the earlier results reported by Choudhary et al. [14], Vyas et al. [24], and Chrungu et al. [25].

Data on morphological characters, yield attributes, and seed yield per plant in C_2   _plants along with parental genotypes are presented in Table 1. Results revealed that C_2_ plants exhibited wider ranges with higher coefficients of variation (CV%) for all the characters studied over the parents. C_2  _plants produced taller plants over the parents. Petal length and width were higher than those of the parents and the hybrid. Meng et al. [26] observed taller plant with larger flowers in synthetic *Brassica *hexaploids over their parental genotypes. The vigorous observation of the C_2_ plants might be due to larger genome size in polyploids over their parental genotypes.

Average pollen fertility in the C_2_ plants was slightly lower than that of both parents, but most of the C_2_ plants (73%) had comparatively higher pollen fertility. The fertility of pollens was also reflected in siliqua setting, that is, those C_2_ plants that had higher percentages of pollen fertility had also higher percentages of siliqua setting. Some C_2_ plants (27%) had lower pollen fertility which might be due to development of aneuploid seeds from C_1_. Number of siliquae per plant in C_2_ plants usually counted as the most important seed yield component was found to be lower in number than the parents. Number of seeds per siliqua, another important component of yield, was lower than Binasarisha-6 but higher than Alboglabra-1. Mean weight of 1000-seed in C_2_ plants was higher than that in the parents. Higher 1000-seed weight was observed in the C_2_ plants which might be due to lower number of siliquae per plant as these two component characters are compensating to each other. Finally, C_2_ plants produced higher mean seed yield per plant than both parents. Siliqua length as well as beak length in C_2_ plants was longer than that of both parents. Although flowering time (50% flowering) in C_2_ plants was earlier than both parents, however the days to maturity were little higher over the early maturing *B. rapa* parent. Wider variation for most of the characters in C_2_ plants might be due to presence of some aneuploids along with euploids. Richharia [27] and Howard [28] reported lower seed setting in artificially developed *Raphanobrassica*. Tokumasu [29] observed wide variations in F_3_ *Raphanobrassica* from a single plant progeny for per cent pollen fertility, per cent siliqua setting, and number of seeds per siliqua. Sarla and Raut [6] observed a wide range of variations for morphological as well as yield contributing characters among 40 C_2_ *B. carinata* plants obtained from a single C_1_ plant and reported that those wide variations were due to presence of aneuploids along with the euploids in C_2_. Formation of univalents or multivalents in C_2_ plants may have contributed to unequal segregation at anaphase-I of meiosis and consequently leaded to a decrease in pollen fertility [30, 31]. Aneuploid formation in the synthetic *B. napus* might be occurred due to affinity of allosyndetic pairing between A and C genomes as reported by Inomata [32], Ahmad et al. [33], and Tian et al. [34] resulting in multivalent association at diakinesis and metaphase-I of meiosis [4, 6, 25, 35].

The results of this study clearly showed that it needs further screening and evaluation for the synthesized *B. napus* in the subsequent generations through selection of desirable genotypes having increased pollen fertility as well as high fruit and seed setting resulting in higher seed yield and desired yield contributing characters. However, more research works are needed to stabilize the synthesized *B. napus*.

## Figures and Tables

**Table 1:** Morphological characters, yield attributes, and seed yield of synthetic *B. napus* and its parental genotypes, Alboglabra-1, and Binasarisha-6.

Characters		Alboglabra-1	Synthetic *B. napus *	Binasarisha-6
Plant height (cm)	Mean	111	143	106
	Range	98–123	132–160	91–116
	CV(%)	6.3	8.3	6.8

Petal length (cm)	Mean	1.9	2.0	1.1
	Range	1.8–2.0	1.7–2.1	1.0–1.2
	CV(%)	2.2	3.0	2.0

Petal width (cm)	Mean	1.17	1.11	0.42
	Range	1.13–1.24	0.95–1.21	0.38–0.47
	CV(%)	3.2	4.6	3.3

Primary branches per plant (no.)	Mean	3.21	4.2	6.8
	Range	2.0–4.0	3–6	5–8
	CV(%)	10.0	13.8	11.3

Pollen fertility (%)	Mean	90	87	91
	Range	87–93	74–94	89–94
	CV(%)	3.0	5.2	3.5

Siliqua setting (%)	Mean	95	93	95
	Range	93–98	71–97	93–99
	CV(%)	2.8	6.4	2.7

Siliqua length (cm)	Mean	5.9	7.7	4.1
	Range	5.3–6.4	6.9–8.1	3.6–4.7
	CV(%)	4.8	10.2	6.0

Beak length (cm)	Mean	0.75	3.02	1.53
	Range	0.69–0.83	2.80–3.23	1.29–1.69
	CV(%)	9.0	12.0	9.9

Siliquae per plant (no.)	Mean	102	77	98
	Range	78–117	61–101	84–110
	CV(%)	9.1	22.4	9.7

Sterile seeds per siliqua (no.)	Mean	—	2.03	—
	Range	—	0.0–3.1	—
	CV(%)	—	11	—

Seeds per siliqua (no.)	Mean	15.6	22.5	22.1
	Range	13–17	17–25	19–25
	CV(%)	7.7	10.4	8.7

1000-seed wt. (g)	Mean	3.2	3.8	3.1
	Range	3.0–3.3	3.7–4.0	3.0–3.3
	CV(%)	3.0	3.1	2.8

Seed yield per plant (g)	Mean	4.9	6.6	6.4
	Range	3.6–6.0	4.3–7.8	5.0–7.4
	CV(%)	11.0	15.8	9.7

Days to 50% flowering	Mean	45	33	35

Days to maturity	Mean	118	95	92
